# Eco-Friendly Selenium-Hyaluronic Acid Nanoconjugates with Potent Anticancer, Antimicrobial, Anti-Inflammatory and Wound-Healing Activities

**DOI:** 10.3390/polym18111376

**Published:** 2026-06-01

**Authors:** Husam Qanash, Bandar Alharbi, Abdulrahman S. Bazaid, Ghaida Alsaif, Talal Alharazi, Naif K. Binsaleh

**Affiliations:** 1 Department of Medical Laboratory Science, College of Applied Medical Sciences, University of Ha’il, Hail 55476, Saudi Arabia; 2 Medical and Diagnostic Research Center, University of Ha’il, Hail 55473, Saudi Arabia

**Keywords:** nanobiocomposites, tissue repair, selenium nanoparticles, anticancer activity, anti-inflammatory activity, antimicrobial activity, wound healing, hyaluronic acid

## Abstract

Cancer and multidrug-resistant microbial infections remain major global health challenges, underscoring the need for multifunctional, biocompatible, and environmentally sustainable therapeutic platforms. Herein, selenium–hyaluronic acid nanoconjugates (Se/HA NPs) were synthesized through an eco-friendly ascorbic acid-mediated reduction approach to improve the bio-functional stability and therapeutic performance of selenium-based nanomaterials. The formation of Se/HA NPs was confirmed by transmission electron microscopy (TEM), X-ray diffraction (XRD), energy-dispersive X-ray spectroscopy (EDX), and Fourier-transform infrared spectroscopy (FTIR). FTIR analysis supported the involvement of ascorbic acid- and hyaluronic acid-associated functional groups in nanoparticle formation and stabilization. TEM revealed well-dispersed, predominantly spherical nanoparticles with diameters ranging from 29.72 to 80.38 nm, while XRD confirmed their crystalline nature with an average crystallite size of 31.2 nm. Biologically, Se/HA NPs exhibited strong antibacterial activity against *Enterococcus faecalis* (21 mm), *Staphylococcus aureus* (24 mm), *Escherichia coli* (25 mm), and *Klebsiella pneumoniae* (27 mm), outperforming hyaluronic acid alone and showing activity comparable to standard antibiotics, with a minimum inhibitory concentration (MIC) of 15.62 µg/mL. Notably, Se/HA NPs showed pronounced antifungal activity against Candida albicans, with an inhibition zone of 34 mm and an MIC of 7.8 µg/mL. In MG-63 osteosarcoma cells, Se/HA NPs demonstrated potent cytotoxicity, with a half-maximal inhibitory concentration (IC_50_) of 8.36 µg/mL compared with 746.37 µg/mL for hyaluronic acid. Moreover, Se/HA NPs enhanced wound closure to 73.41% and showed strong anti-inflammatory activity, with an IC_50_ of 5.37 µg/mL, demonstrating multifunctional bioactivity.

## 1. Introduction

The development of nanoparticles represents one of the most important advances in modern nanotechnology, bridging fundamental science with diverse industrial, environmental, and biomedical applications [1]. Over the past decade, considerable attention has been directed toward nanoparticles composed of elements such as copper, selenium, zinc, and titanium because of their distinctive physicochemical and biological properties [2,3]. More recently, research has shifted from the synthesis of single-component nanoparticles toward the design of multifunctional nano-systems with controlled surface characteristics, improved stability, and enhanced biological performance. It is important to note that the discussion presented herein focuses specifically on inorganic nanoparticles, including elemental selenium nanoparticles, as well as their hybrid organic-inorganic nanocomposite systems. Surface modification with polymers, natural biomolecules, or bioactive ligands can improve nanoparticle dispersibility, biocompatibility, targeting ability, and interaction with biological systems, thereby increasing their usefulness in drug delivery, antimicrobial therapy, tissue repair, and other biomedical applications. In this context, hybrid nanocomposites that integrate organic and inorganic components have demonstrated improved functional performance in several fields, including wastewater treatment, catalysis, tissue engineering, and biomedical material development [4,5,6,7].

Within the category of inorganic nanomaterials, selenium nanoparticles (Se NPs) have attracted substantial interest because selenium is an essential trace element involved in antioxidant defense, immune regulation, redox balance, and cellular homeostasis [8]. Compared with conventional selenium forms, Se NPs are generally considered to have improved bioavailability, higher biological activity, and a wider safety margin, making them promising candidates for dietary, antioxidant, and biomedical applications. Over the past ten years, Se NPs have been successfully explored as antioxidant agents, nutritional supplements, and diagnostic or therapeutic nanomaterials with reduced toxicity compared with selenium salts. However, conventional commercial approaches for producing inorganic Se NPs often require complex procedures, harsh reaction conditions, high pressure, elevated temperature, or chemical reducing agents that may raise environmental and safety concerns [9,10]. These limitations have encouraged the development of greener and more sustainable synthesis strategies capable of controlling nanoparticle size, morphology, stability, and biological activity.

Green synthesis approaches have emerged as attractive alternatives for producing metal and metalloid nanoparticles under milder and more environmentally compatible conditions [11]. Several biological systems and natural molecules, including bacteria, fungi, yeast, and biomolecules, have been used to reduce toxic metal ions into nanoscale particles with defined size and morphology [5,9]. Such approaches can reduce the need for hazardous reagents while simultaneously improving nanoparticle stabilization through naturally derived functional groups. In particular, ascorbic acid is a biocompatible reducing agent with antioxidant properties, making it suitable for the eco-friendly preparation of selenium-based nanostructures [12]. Its use may also contribute to nanoparticle stabilization by participating in surface interactions during nanoparticle formation.

Selenium nanoparticles are especially promising in biomedical research because of their strong antioxidant activity, relatively low toxicity, and ability to modulate biological pathways involved in oxidative stress, inflammation, microbial survival, and cancer progression [13,14]. Several studies have reported that Se NPs exhibit anticancer effects against different tumor types, further supporting their therapeutic relevance [10]. For example, Ren et al. [15] evaluated the anticancer effect of Se/HA nanoparticles with a mean size of 50–70 nm in a Heps tumor mouse model and reported a significant reduction in tumor weight compared with the model control group (*p* < 0.05). Hyaluronic acid-modified selenium nanoparticles have also been shown to enhance hepatocellular carcinoma treatment by improving transfection efficiency and cytotoxic activity both in vitro and in vivo. Similarly, Se/HA/PEI nanoparticles have been prepared and evaluated in HepG2 cells and in vivo models [16]. These findings highlight the therapeutic value of combining selenium nanoparticles with biologically active polymers to improve targeting, cellular interaction, and functional efficacy.

Hyaluronic acid (HA) is a naturally occurring biopolymer with excellent biocompatibility, biodegradability, water retention capacity, and biological relevance in tissue repair and extracellular matrix organization [17]. Because HA can interact with CD44 receptors, which are frequently overexpressed in several cancer cells and inflammatory tissues, it is widely used as a functional coating or carrier in targeted nanomedicine [18]. HA modification can improve nanoparticle stability, cellular uptake, and biological selectivity while also supporting wound-healing and tissue-regenerative processes. The Se/HA NPs prepared by direct reduction of seleninic acid in the presence of HA showed strong free-radical scavenging activity and efficient astrocyte internalization through CD44 receptor-mediated interaction [19]. These findings suggest that HA-functionalized Se NPs may provide a biologically favorable platform for developing multifunctional therapeutic nanomaterials.

Several biopolymers have been explored for the conjugation of selenium nanoparticles, including chitosan, alginate, gelatin, and hyaluronic acid [15,16,19]. Among these, hyaluronic acid (HA) was selected in the present study for multiple reasons. First, HA exhibits exceptional biocompatibility and biodegradability, as it is a natural component of the extracellular matrix. Second, HA can specifically bind to CD44 receptors, which are overexpressed on various cancer cells and inflamed tissues, enabling targeted drug delivery. Third, HA possesses inherent wound-healing and anti-inflammatory properties that can synergize with the bioactivity of selenium. Previous studies have reported Se/HA nanocomposites prepared by different methods. For instance, Ren et al. [15] synthesized Se/HA nanoparticles using sodium selenite and HA under acidic conditions, achieving particles of 50–70 nm with antitumor activity. Luo et al. [19] prepared HA-functionalized Se NPs for spinal cord injury repair via CD44-mediated internalization. However, most reported methods require harsh conditions or complex procedures.

Despite these advances, limited studies have investigated eco-friendly Se/HA nanoconjugates as integrated multifunctional agents with simultaneous anticancer, antimicrobial, antifungal, anti-inflammatory, and wound-healing activities. This gap is particularly important because cancer, chronic wounds, inflammation, and multidrug-resistant microbial infections are often interconnected clinical challenges that require therapeutic materials with broad biological functionality. Therefore, developing a simple, green, and biologically active Se/HA nanocomposite may provide a promising approach for biomedical applications where antimicrobial protection, inflammation control, tissue repair, and anticancer activity are needed within a single nanosystem.

In the present study, selenium/hyaluronic acid nanocomposites (Se/HA) were synthesized using vitamin C, also known as ascorbic acid, as an eco-friendly reducing and stabilizing agent. The physicochemical characteristics of the synthesized Se/HA nanocomposites, including morphology, elemental composition, functional groups, and crystallinity, were investigated using advanced analytical techniques. The biological performance of the nanocomposites was then evaluated against multidrug-resistant bacteria, *Candida albicans*, and MG63 osteosarcoma cells. In addition, the anti-inflammatory and wound-healing properties of HA and Se/HA were assessed to determine whether hyaluronic acid conjugation could enhance the therapeutic potential of selenium-based nanomaterials. By integrating green synthesis, HA-mediated functionalization, and multiple biomedical assays, this study aims to introduce Se/HA nanoconjugates as a sustainable and multifunctional nanoplatform with promising potential for antimicrobial protection, cancer therapy, inflammation modulation, and tissue repair. The advantages of the proposed Se/HA nanostructures over existing systems include: (i) a green, one-pot synthesis using ascorbic acid as a biocompatible reducing agent, thereby avoiding toxic chemicals; (ii) well-controlled spherical morphology with a narrow size distribution of 29.7–80.4 nm; (iii) high colloidal stability, as indicated by a zeta potential of −32.7 mV; (iv) multifunctional bioactivity, including anticancer, antimicrobial, anti-inflammatory, and wound-healing effects, within a single nanoplatform; and (v) high yield of 86.5% under mild aqueous conditions. These features make the Se/HA NPs a more sustainable and versatile alternative to previously reported systems.

## 2. Materials and Methods

### 2.1. Materials

Sodium selenite (Na_2_SeO_3_); ascorbic acid, also known as vitamin C; and hyaluronic acid (HA) were purchased from Sigma-Aldrich (St. Louis, MO, USA) with a stated purity of ≥97% and a molecular weight of approximately 1.5–1.8 MDa, corresponding to high-molecular-weight HA, as confirmed by the certificate of analysis. All chemicals were of analytical grade and were used as received without further purification.

### 2.2. Preparation of Se/HA NPs

Selenium-hyaluronic acid nanoparticles (Se/HA NPs) were prepared using an ascorbic acid-mediated reduction method with slight modification [20]. Briefly, 5 mL of ascorbic acid solution (4 mM) was added dropwise to 5 mL of sodium selenite (Na_2_SeO_3_) solution (1 mM) under continuous stirring. The reaction mixture was stirred for 4 h to allow the reduction of selenite ions and the formation of Se NPs. Subsequently, formed Se NPs were conjugated with HA by adding 5 mL of HA solution (4 mM), previously dissolved in deionized water, followed by continuous stirring for an additional 4 h to promote Se/HA nanoconjugate formation. The resulting suspension was centrifuged at 4000 rpm for 20 min, and the nanoparticles were washed three times with deionized water, with each wash consisting of resuspension followed by centrifugation at 4000 rpm for 20 min, to remove unreacted sodium selenite, ascorbic acid, and free hyaluronic acid. Deionized water was used exclusively for washing. The pH of the reaction mixture was adjusted separately using 0.1 M NaOH or 0.1 M HCl, as needed, to maintain a pH of 6.8–7.2 during synthesis. Finally, the purified Se/HA NPs were lyophilized to obtain a red powder with a yield of 86.5%. The pH of the reaction mixture was monitored throughout the synthesis and maintained at 6.8–7.2 using a calibrated pH meter. The HA concentration of 4 mM was optimized in preliminary experiments. HA concentrations below 2 mM resulted in incomplete nanoparticle coating and visible aggregation within 24 h, whereas concentrations above 6 mM led to excessive viscosity and difficulty in purification. Therefore, an HA concentration of 4 mM was selected as optimal because it provided complete surface coverage, prevented aggregation, and maintained colloidal stability, with PDI values <0.25 for at least 14 days.

The stabilization of Se/HA NPs is achieved through two complementary mechanisms: (i) electrostatic stabilization provided by the negatively charged carboxylate (−COO^−^) and hydroxyl (−OH) groups of hyaluronic acid, which create a zeta potential of −32.7 mV and induce repulsive forces that prevent particle aggregation; and (ii) steric stabilization imparted by the long polymeric chains of HA, which physically hinder nanoparticle proximity and agglomeration. The combination of these effects ensures long-term colloidal stability without the need for synthetic surfactants.

### 2.3. Characterization of Se/HA NPs

The physicochemical characteristics of the synthesized Se/HA NPs were investigated using multiple analytical techniques. The morphology, size distribution, and particle shape were examined by transmission electron microscopy (TEM; JEM-2100 PLUS, JEOL, Tokyo, Japan). Elemental composition was analyzed using energy-dispersive X-ray spectroscopy (EDX) coupled with a field-emission scanning electron microscope (JSM-7600F, JEOL, Tokyo, Japan). The crystalline phase and structural properties of the produced nanoparticles were evaluated by X-ray diffraction (XRD) using a Bruker D8 Discover X-ray diffractometer (Bruker, Billerica, MA, USA). Fourier-transform infrared spectroscopy (FTIR) was performed to identify the functional groups involved in nanoparticle formation and HA conjugation. FTIR spectra of HA and the Se/HA nanocomposite were recorded using a Thermo Scientific Nicolet iS50 FTIR spectrometer (Thermo Scientific, Waltham, MA, USA) with KBr pellets, as previously described [21]. The hydrodynamic diameter, polydispersity index (PDI), and zeta potential of the synthesized Se/HA NPs were determined using dynamic light scattering (DLS) and electrophoretic light scattering (Zetasizer Nano ZS, Malvern Panalytical, Malvern, UK). Briefly, lyophilized Se/HA NPs were dispersed in deionized water (0.1 mg/mL) and sonicated for 15 min to ensure uniform dispersion. Measurements were performed in triplicate at 25 °C using disposable capillary cuvettes. The zeta potential was calculated from electrophoretic mobility using the Smoluchowski equation.

To evaluate the time-dependent colloidal stability of Se/HA NPs, the nanoparticles were dispersed in deionized water (0.1 mg/mL) and stored at 4 °C for 30 days. At predetermined time intervals of 0, 7, 14, 21, and 30 days, aliquots were withdrawn and analyzed for hydrodynamic diameter, PDI, and zeta potential using the Zetasizer Nano ZS, as described above. Visual inspection for agglomeration, precipitation, or color change was also performed. All measurements were conducted in triplicate.

### 2.4. Antimicrobial Activity: Agar Diffusion, MIC, MBC, and MFC Assays

The antimicrobial activity of HA and Se/HA NPs was evaluated against selected bacterial and fungal strains using agar well diffusion and broth microdilution assays. These assays were performed to determine the inhibition zone diameter, minimum inhibitory concentration (MIC), minimum bactericidal concentration (MBC), and minimum fungicidal concentration (MFC). Initial antimicrobial screening was conducted using agar well diffusion method. Sterile Mueller-Hinton agar plates were uniformly inoculated with standardized microbial suspensions of the test organisms, including *Enterococcus faecalis* (ATCC 29212), *Staphylococcus aureus* (ATCC 6538), *Escherichia coli* (ATCC 8739), *Klebsiella pneumoniae* (ATCC 13883), *Pseudomonas aeruginosa* (ATCC 90274), and *Candida albicans* (ATCC 10221). Wells of 6 mm diameter were aseptically prepared using a sterile cork borer, and 100 µL of each test sample was added to the wells at the required concentrations. The plates were incubated at 35 ± 2 °C for 16–24 h. Antimicrobial activity was then assessed by measuring the diameter of the inhibition zone around each well in millimeters [22]. For quantitative assessment of antimicrobial potency, MIC values were determined using the broth microdilution method according to CLSI M07 [23] and ISO 20776-1 guidelines [24]. Serial two-fold dilutions of each sample were prepared in sterile 96-well microtiter plates using Tryptic Soy Broth, with concentrations ranging from 1000 to 1.95 µg/mL. Fresh microbial suspensions were prepared from 18-h cultures and adjusted to a turbidity equivalent to the 0.5 McFarland standard, corresponding to approximately 2 × 10^8^ CFU/mL. The suspensions were then further diluted to obtain a final inoculum of approximately 5 × 10^5^ CFU/mL in each well. Each well contained the test sample dilution and microbial inoculum. Growth control wells without antimicrobial agents and sterility control wells without inoculation were included in each experiment. The microtiter plates were incubated at 35 ± 2 °C for 16–20 h. Microbial growth was evaluated visually and spectrophotometrically at 630 nm using a BioTek 800 TS microplate reader (Biotek, Winooski, VT, USA). The MIC was defined as the lowest concentration of the tested sample that completely inhibited visible microbial growth, as indicated by the absence of turbidity compared with the growth control. Following MIC determination, MBC values were assessed by subculturing aliquots from wells corresponding to the MIC and higher concentrations onto fresh agar plates. After incubation at 35 ± 2 °C for 24 h, colony-forming units were counted. The MBC was defined as the lowest concentration that produced a ≥99.9% reduction in viable bacterial count compared with the initial inoculum, thereby confirming bactericidal activity. For *C. albicans*, MFC values were determined using the same microdilution approach. Aliquots from clear wells showing no visible fungal growth were sub-cultured onto Sabouraud Dextrose Agar and incubated at 30 °C for 48 h. The MFC was defined as the lowest concentration showing complete absence of fungal growth after subculture, indicating fungicidal activity [25].

### 2.5. Evaluation of Anticancer Activity

The antiproliferative activity of HA and Se/HA samples was evaluated in vitro using human osteosarcoma MG-63 cancer cells (VACSERA, Cairo, Egypt). This assay was performed to determine the cytotoxic potential of the synthesized nanoconjugates and to assess their concentration-dependent effect on tumor cell viability. MG-63 cells were cultured in appropriate growth medium supplemented with fetal bovine serum and antibiotics, then maintained in a humidified incubator at 37 °C with 5% CO_2_. For the cytotoxicity assay, cells were seeded into 96-well plates at a uniform density and allowed to adhere overnight to ensure stable attachment before treatment. Stock solutions of the tested samples were prepared and serially diluted in culture medium to obtain final concentrations ranging from 31.25 to 1000 µg/mL. After removal of the old growth medium, cells were treated with the prepared concentrations of HA and Se/HA and incubated for 24–48 h. Untreated cells were included as the control group and considered to represent 100% cell viability. Cell viability was assessed using the colorimetric MTT assay, which is based on the ability of metabolically active cells to reduce MTT into insoluble purple formazan crystals. After treatment, MTT solution was added to each well and incubated under standard culture conditions. The formed formazan crystals were then dissolved using a suitable solubilizing agent, and absorbance was measured spectrophotometrically. The percentage of viable cells was calculated relative to untreated control cells, as previously described [22]. Cytotoxicity was expressed as the reduction in cell viability after treatment, and the antiproliferative potency of each sample was determined based on the half-maximal inhibitory concentration (IC_50_).

The percentage of cytotoxicity was calculated using the following equation:

$$\mathrm{Cytotoxicity}\;(\%)=\left(1-\frac{\mathrm{Absorbance}\;\mathrm{of}\;\mathrm{treated}\;\mathrm{cells}}{\mathrm{Absorbance}\;\mathrm{of}\;\mathrm{control}\;\mathrm{cells}}\right)\times 100$$

The IC_50_ value was determined from the dose–response curve and defined as the concentration required to inhibit 50% of MG-63 cell viability compared with untreated control cells.

### 2.6. Scratch Wound-Healing Assay

The migratory capacity of HFB4 cells (VACSERA, Cairo, Egypt) under different treatment conditions was evaluated using the scratch wound-healing assay. Cells were seeded into 6-well culture plates and incubated under standard culture conditions until a complete and uniform confluent monolayer was formed in each well. Establishing comparable confluency before initiating the assay was essential to ensure consistency and reproducibility across all experimental groups. A straight scratch was then created in each well using a sterile yellow pipette tip held at an approximately 30° angle to generate a controlled and relatively uniform wound gap. After scratching, the wells were gently washed with phosphate-buffered saline (PBS) to remove detached cells and cellular debris, leaving a clear cell-free area that simulated a wound region suitable for monitoring cell migration. The experiment included three groups: an untreated control group of HFB4 cells, an HA-treated group, and a group treated with Se/HA nanoparticles at a concentration of 31.25 µg/mL. Immediately after scratch formation and washing, the respective treatments were added to the assigned wells, and the cells were incubated under appropriate culture conditions. Images of the scratched area were captured immediately after wound creation (0 h) and after 48 h of incubation using an inverted light microscope (Nikon Instruments Inc., Nikon Healthcare, Melville, NY, USA) equipped with a 10× objective lens [26]. Cell migration was assessed by measuring the reduction in wound width over time. The rate of migration (RM) was calculated using the following equation:

$$RM=\frac{W_{i}-W_{f}}{t}$$

where $W_{i}$ represents the initial average wound width at 0 h, $W_{f}$ represents the final average wound width after incubation, and t represents the time interval in hours. The rate of migration was calculated using the following equation:

$$Wound\;Closure\;(\%)=\frac{A_{t=0}-A_{t=\Delta t}}{A_{t=0}}\times 100$$

where $A_{t=0}$ represents the initial wound area measured immediately after scratching, and $A_{t=\Delta t}$ represents the wound area after 48 h of incubation. Furthermore, the absolute area difference was determined as the subtraction between initial and final wound areas (Ai–Af) to further quantify wound reduction.

### 2.7. Anti-Inflammatory Activity (Protein Denaturation Assay) via Protein Denaturation

The anti-inflammatory activity of HA and Se/HA nanoparticles was evaluated using the bovine serum albumin (BSA) protein denaturation inhibition assay and compared with the standard anti-inflammatory drug diclofenac sodium. The assay was performed with slight modifications to previously reported methods [27]. Briefly, serial concentrations of each sample were prepared at 1.56, 3.125, 6.25, 12.5, 25, 50, 100, and 200 µg/mL. In each experimental tube, 50 µL of the test sample was mixed with 450 µL of 1% aqueous BSA solution. The pH of the reaction mixture was carefully adjusted to 6.3 using 1 N HCl, as mildly acidic conditions promote controlled protein denaturation and improve assay reproducibility. The reaction mixtures were first incubated at room temperature for 20 min to allow interaction between the tested compounds and protein molecules. Subsequently, the samples were heated in a water bath at 55 °C for 30 min to induce thermal protein denaturation. After heating, the samples were allowed to cool to room temperature before spectrophotometric measurement. An untreated BSA solution served as the control and represented maximum protein denaturation. The degree of protein denaturation was determined by measuring absorbance at 670 nm using a Biosystem 310 Plus spectrophotometer (Biosystems, Barcelona, Spain). Mean absorbance values and standard deviations were calculated for each concentration. The percentage inhibition of protein denaturation was calculated using the following equation:

$$Inhibition\;(\%)=\frac{A_{control}-A_{sample}}{A_{control}}\times 100$$

where $A_{control}$ represents the absorbance of the control sample, and $A_{sample}$ represents the absorbance of the tested material. Dose-dependent inhibition curves were generated for all tested samples, and IC_50_ values were calculated from the plotted concentration-response data to compare the anti-inflammatory potency of HA, Se/HA nanoparticles, and diclofenac sodium. Lower IC_50_ values were interpreted as stronger inhibition of protein denaturation and, consequently, greater in vitro anti-inflammatory activity.

### 2.8. Statistical Analysis

All experiments were performed in triplicate (n = 3), with three independent biological replicates. Results are expressed as mean ± standard deviation (SD). All statistical analyses were performed using Minitab 18. Quantitative data were analyzed using one-way analysis of variance followed by Tukey’s post hoc test for multiple comparisons when appropriate. Data were considered statistically significant at a probability level of *p* < 0.05. Results were expressed as mean ± standard deviation where applicable.

## 3. Results and Discussion

### 3.1. Morphological Characterization of Se/HA Nanoparticles

**Figure 1:**
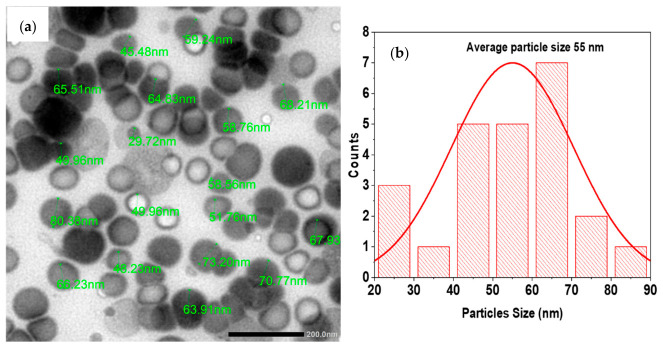
Transmission electron microscopy (TEM) characterization of Se/HA nanoparticles. (**a**) TEM image showing the morphology of Se/HA nanoparticles. (**b**) Particle size distribution of the synthesized Se/HA nanoparticles.

The synthesized Se/HA nanoparticles were characterized using TEM, EDX, XRD, and FTIR to confirm their morphology, elemental composition, crystalline structure, and surface functionalization. TEM analysis revealed that the Se/HA nanoparticles were predominantly monodisperse and spherical, with particle sizes ranging from 29.72 to 80.38 nm and an average size of approximately 55 nm (Figure 1a,b). The hydrodynamic properties of Se/HA NPs were further characterized by DLS. The average hydrodynamic diameter was determined to be 68.4 ± 4.2 nm, which was slightly larger than the TEM-derived size of approximately 55 nm, likely due to the hydration layer and the presence of the HA corona surrounding the nanoparticles. The polydispersity index (PDI) was measured as 0.21 ± 0.03, indicating a moderately narrow size distribution and good colloidal homogeneity. Zeta potential analysis revealed a negative surface charge of −32.7 ± 2.8 mV for Se/HA NPs. This negative zeta potential is attributed to the abundant carboxylate (−COO^−^) and hydroxyl (−OH) groups of hyaluronic acid on the nanoparticle surface. The measured magnitude of the zeta potential (>30 mV) suggests good electrostatic stabilization, which helps prevent nanoparticle aggregation and enhances colloidal stability in aqueous media.

The nanoparticles were prepared through a simple reduction approach in which water-soluble ascorbic acid acted as a reducing and stabilizing agent, while HA served as a capping matrix that supported nanoparticle formation and minimized aggregation. This protective role of HA is particularly important because polymeric capping can improve colloidal stability and maintain nanoscale particle distribution by preventing uncontrolled particle growth and agglomeration [28]. TEM images further suggested the formation of a core–shell-like structure, indicating successful association between selenium and the HA coating (Figure 1a). Comparable findings have been reported by Zhang et al. [29], who synthesized selenium nanoparticles from sodium selenite using gum arabic (a tree gum) as a stabilizer and obtained particles with an average diameter of 70.9 nm. Collectively, these findings demonstrate that the ascorbic acid-assisted synthesis strategy, combined with HA capping, produced stable, spherical, nanosized Se/HA particles with a core–shell morphology, providing a structurally controlled nanoplatform that supports the novelty of this study and its relevance for multifunctional biomedical applications.

### 3.2. Elemental Composition and Crystalline Structure of Se/HA Nanoparticles

**Figure 2:**
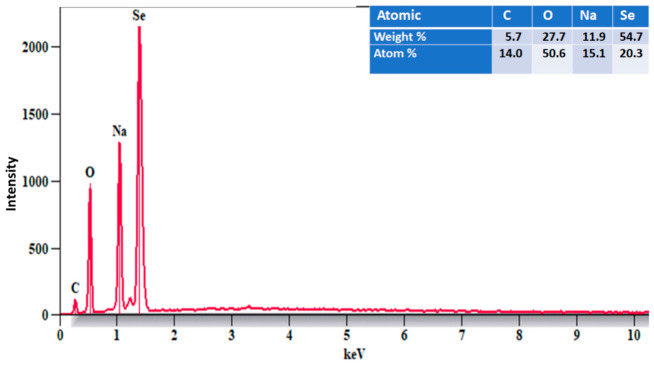
Energy-dispersive X-ray spectroscopy pattern of the synthesized Se/HA nanoparticles.

**Figure 3:**
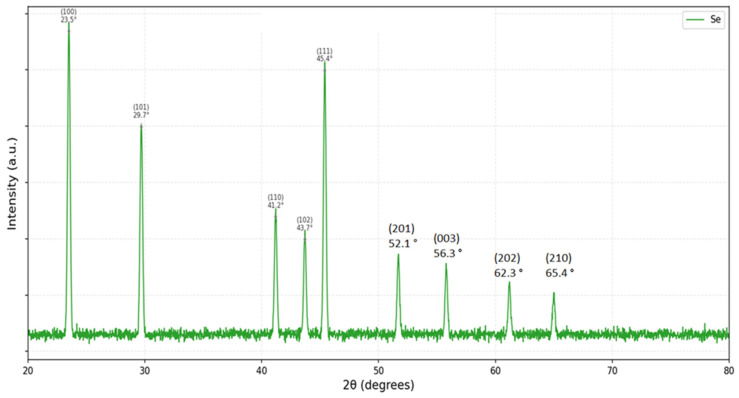
X-ray diffraction (XRD) pattern of the green-synthesized Se/HA nanoparticles.

Energy-dispersive X-ray spectroscopy analysis confirmed the elemental composition of the synthesized Se/HA nanoparticles. As shown in Figure 2, the EDX spectrum displayed a strong selenium signal at approximately 1.3 keV, confirming the successful incorporation of selenium into the Se/HA nanostructure. A sodium-related peak was also observed, which may be attributed to residual sodium from the precursor salt or to the glass substrate used during analysis [30]. The carbon and oxygen signals were associated with the organic components present on the nanoparticle surface, particularly ascorbic acid and HA, which likely contributed to nanoparticle reduction, capping, and stabilization. The crystalline phase of the produced Se/HA nanoparticles was further examined using X-ray diffraction (XRD), as presented in Figure 3. The XRD pattern showed characteristic diffraction peaks at 2θ = 23.5°, 29.7°, 41.2°, 43.7°, 45.4°, 52.1°, 56.3°, 62.3°, and 65.4°, corresponding to the 100, 101, 110, 102, 111, 201, 003, 202, and 210 planes of trigonal selenium, respectively, with lattice parameters of c = 4.954 Å and a = 4.366 Å, consistent with JCPDS card no. 06-0362 [31,32]. The broad baseline observed in the XRD profile suggests the presence of a minor amorphous component, estimated at approximately 15%, which may be related to HA coating or surface-associated organic residues. The average crystallite size of the Se/HA nanoparticles was calculated using the Scherrer equation: D = k λ/β cosθ, where D represents the crystallite size, k is the shape factor with a value of 0.9, λ is the incident X-ray wavelength, β is the full width at half maximum of the diffraction peak, and θ is the diffraction angle. Based on this equation, the average crystallite size was estimated to be 31.2 nm, confirming the nanoscale crystalline nature of the synthesized particles. No additional impurity phases were detected beyond the assigned selenium reflections, supporting the structural purity of the prepared Se/HA nanoparticles. Together, the EDX and XRD findings confirm that the eco-friendly ascorbic acid-assisted approach successfully produced selenium-rich, crystalline Se/HA nanostructures with organic surface stabilization, highlighting the novelty of this work in generating a structurally defined and bio-functional selenium-HA nanoplatform.

Furthermore, the zeta potential of Se/HA NPs was measured as −32.7 ± 2.8 mV, confirming a stable negatively charged surface imparted by the HA coating. The PDI value of 0.21 ± 0.03 reflects a relatively homogeneous nanoparticle population with a low aggregation tendency. These findings are consistent with the TEM observations of well-dispersed spherical nanoparticles and support the role of HA as an effective capping and stabilizing agent. The discrepancy between the TEM-derived particle size, approximately 55 nm, and the XRD-derived crystallite size, 31.2 nm, is expected for polymer-coated nanoparticles. XRD detects only the crystalline selenium core, as calculated using the Scherrer equation, whereas TEM visualizes the entire nanoparticle, including the amorphous hyaluronic acid shell. Therefore, the HA shell thickness is estimated to be approximately 12 nm, which is consistent with the core–shell morphology observed (Figure 1a).

#### Time-Dependent Colloidal Stability of Se/HA NPs

The colloidal stability of Se/HA NPs was monitored over 30 days of storage at 4 °C, and the results are summarized in Supplementary Table S1. Freshly prepared Se/HA NPs at day 0 exhibited an average hydrodynamic diameter of 68.4 ± 4.2 nm, a PDI of 0.21 ± 0.03, and a zeta potential of −32.7 ± 2.8 mV. After 7 days, no significant changes were observed in any of the three parameters (*p* > 0.05). At day 14, a slight but statistically insignificant increase in diameter (72.3 ± 5.8 nm) and PDI (0.24 ± 0.05) was noted. By day 21, the hydrodynamic diameter had increased significantly to 78.6 ± 6.5 nm, and the PDI had increased to 0.28 ± 0.06 (*p* < 0.05 compared with day 0), indicating the onset of minor aggregation. The zeta potential remained above −28 mV. After 30 days, further increases were observed in hydrodynamic diameter and PDI values, reaching 89.2 ± 8.1 nm and 0.34 ± 0.07, respectively, while the zeta potential decreased to −25.4 ± 4.2 mV, confirming progressive aggregation. Visual inspection showed no precipitation over 30 days, although slight turbidity was noted at day 30. These results indicate that Se/HA NPs remain stable for up to 14–21 days under refrigerated storage conditions.

### 3.3. FTIR Confirmation of HA-Mediated Surface Functionalization

**Figure 4:**
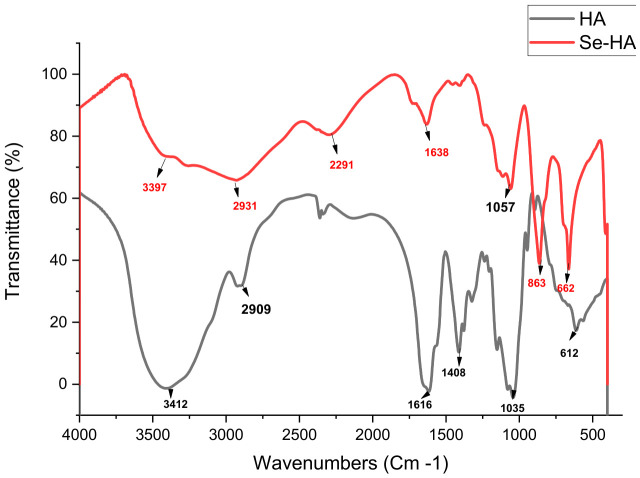
Fourier-transform infrared spectroscopy analysis of HA and Se/HA nanoparticles.

FTIR analysis was performed to identify the functional groups involved in the formation and stabilization of the Se/HA nanocomposite. The FTIR spectrum of HA exhibited intense peaks at 3412, 2909, 1622, 1035, and 612 cm^−1^, which shifted to 3397, 2931, 1638, 1037, and 662 cm^−1^, respectively, after Se/HA nanoparticle formation (Figure 4). Notably, the Se/HA spectrum exhibited additional peaks at 2291 cm^−1^ and 863 cm^−1^ that were absent in pure HA. The peak at 2291 cm^−1^ is attributed to the −SH stretching vibration of thiol-containing intermediates or to overtones of C≡N or C≡C bonds, possibly arising from ascorbic acid degradation products. The peak at 863 cm^−1^ may correspond to the C–O–C stretching vibration of oxidized ascorbic acid, dehydroascorbic acid, or to Se–O vibrations. These peaks suggest that ascorbic acid not only reduces selenite but also undergoes partial oxidation, with its byproducts adsorbing onto the nanoparticle surface. This finding confirms the active participation of ascorbic acid in surface stabilization, beyond its role as a reducing agent.

The broad band observed at 3412 cm^−1^ in native HA is attributed to O–H stretching vibrations and N-H stretching vibrations associated with the N-acetyl side chain. The moderately intense overlapping bands around 2909 cm^−1^ correspond to C–H stretching vibrations. In addition, the bands at 1622 and 1418 cm^−1^ are assigned to the asymmetric C=O and symmetric C–O stretching modes of carboxyl groups in hyaluronate [33]. The absorption band at 1035 cm^−1^ is related to the primary alcohol C–O stretching vibration [34]. The observed shifts in peak position, together with changes in peak intensity after selenium incorporation, indicate the participation of HA functional groups in the capping and stabilization of Se/HA nanoparticles. HA does not participate in the reduction of selenite ions, which is accomplished entirely by ascorbic acid. Instead, HA acts as a capping and stabilizing agent after Se NP formation, contributing to nanoparticle stabilization through its functional groups. Moreover, the appearance and modification of characteristic peaks following the interaction between HA and selenium further support successful conjugation between HA and Se NPs (Figure 4). In particular, the distinct band at 2931 cm^−1^ after Se NP loading provides additional evidence of HA attachment to the nanoparticle surface, consistent with previous findings [35]. Overall, FTIR analysis confirms that HA was not merely physically mixed with selenium but actively participated in nanocomposite formation, supporting the novelty of this study by demonstrating a green, HA-stabilized Se nanoplatform with surface chemistry suitable for enhanced biological performance. Regarding the chemical bond formed between Se NPs and HA, no new covalent bond, such as Se–C or Se–O–C, was detected in the FTIR spectrum. This is consistent with the literature on polymer-coated selenium nanoparticles [35,36]. Instead, the interaction is primarily non-covalent, involving hydrogen bonding between the hydroxyl (−OH) and carboxyl (−COOH) groups of HA and the surface of Se NPs, as well as electrostatic interactions between negatively charged HA carboxylate groups and partially positive selenium surface sites. The observed shifts in peak positions, such as the O–H band from 3412 to 3397 cm^−1^ and the C=O band from 1616 to 1638 cm^−1^, together with changes in peak intensity, confirm that HA is not merely physically mixed but is strongly adsorbed onto the Se NP surface through these non-covalent forces. This conclusion is further supported by the high negative zeta potential and the core–shell morphology observed in TEM images. Similar non-covalent stabilization has been reported for polysaccharide-coated Se NPs [29,31].

### 3.4. Enhanced Broad-Spectrum Antimicrobial Activity of Se/HA Nanoparticles

**Figure 5:**
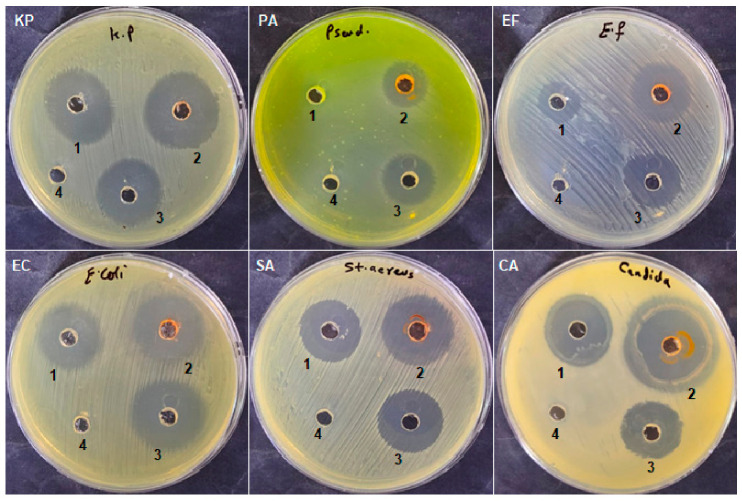
Antimicrobial activity of hyaluronic acid (1), selenium-hyaluronic acid nanoparticles (2), standard drug (3), and negative control (4) against *Escherichia coli* (EC), *Staphylococcus aureus* (SA), *Klebsiella pneumoniae* (KP), *Pseudomonas aeruginosa* (PA), *Enterococcus faecalis* (EF), and *Candida albicans* (CA).

The antimicrobial activity results presented in Table 1 and Figure 5 demonstrate that Se/HA nanoparticles exhibited stronger antimicrobial activity than HA alone and, in several cases, showed inhibition comparable to or greater than the positive controls, gentamicin for bacteria and nystatin for fungi. Against *Enterococcus faecalis*, Se/HA produced an inhibition zone of 21 mm, compared with 14 mm for HA and 18 mm for the antibiotic control, indicating a clear enhancement in antibacterial efficacy after selenium incorporation. Similarly, Se/HA showed the strongest activity against *Staphylococcus aureus*, with an inhibition zone of 24 mm, slightly exceeding the positive control. The MIC and MBC values further supported these observations, as Se/HA required lower concentrations to inhibit and kill bacterial cells compared with HA. Gram-negative bacteria also showed marked susceptibility to Se/HA, particularly *Escherichia coli* and *Klebsiella pneumoniae*, which displayed large inhibition zones of 25 and 27 mm, respectively, comparable to the standard control. Consistently lower MIC values for Se/HA, reaching 15.62 µg/mL, confirm its improved antimicrobial potency. In contrast, *Pseudomonas aeruginosa* showed complete resistance to HA, whereas Se/HA demonstrated moderate inhibition with a 15 mm zone; however, its higher MIC and MBC values indicate comparatively reduced susceptibility of this strain. Notably, Se/HA exhibited pronounced antifungal activity against *Candida albicans*, producing a large inhibition zone of 34 mm, which exceeded both HA (23 mm) and nystatin (22 mm). The MIC and MFC values of Se/HA against *C. albicans* were 7.8 and 15.62 µg/mL, respectively, representing the lowest values among all tested organisms and confirming strong anti-fungal potency. These findings are consistent with Li et al. [36], who reported that a carboxymethyl chitosan/oxidized hyaluronic acid hydrogel exhibited markedly improved antimicrobial activity after incorporation of SeNPs, with inhibition rates of 96.0% against *Staphylococcus aureus* and 91.67% against *Escherichia coli*. Their findings highlight the important contribution of SeNPs to infection control in wound-related applications, particularly chronic diabetic wound management. Overall, the present results show that conjugating selenium nanoparticles with HA substantially enhances antimicrobial performance across bacterial and fungal pathogens, supporting the novelty of Se/HA as a multifunctional nanoplatform with potential relevance for antimicrobial protection, wound infection control, and biomedical material development.

The moderate antimicrobial activity observed for pure HA, with inhibition zones of 14–23 mm, warrants explanation. While native high-molecular-weight HA typically lacks direct bactericidal activity, several factors may account for our observations: (i) the HA used in this study, Sigma-Aldrich, ≥97% purity, may contain trace impurities or endotoxins that contribute to microbial inhibition; (ii) the high concentration of HA, 4 mM, used in the agar diffusion assay may create osmotic effects or physically impede microbial growth; (iii) HA can bind divalent cations, such as Mg^2+^ and Ca^2+^, which are essential for bacterial membrane integrity, indirectly suppressing growth; and (iv) some commercial HA preparations may exhibit minor antimicrobial activity due to residual proteins or manufacturing byproducts. Importantly, the antimicrobial activity of Se/HA NPs was consistently and significantly higher than that of HA alone (*p* ≤ 0.05), confirming that selenium conjugation is responsible for the enhanced effect.

#### Proposed Antimicrobial Mechanism of Se/HA NPs

Although mechanistic assays were beyond the scope of this study, the antimicrobial activity of Se/HA NPs is proposed to involve multiple synergistic pathways based on prior reports [37]. First, Se/HA NPs can generate reactive oxygen species (ROS) upon contact with microbial cells, inducing oxidative damage to lipids, proteins, and DNA. Second, direct nanoparticle–membrane interactions may alter membrane permeability, leading to leakage of intracellular contents. Third, selenium may interfere with microbial selenoprotein synthesis and redox homeostasis by substituting sulfur in essential enzymes. Fourth, Se NPs may inhibit biofilm formation by downregulating quorum-sensing genes. Hyaluronic acid conjugation enhances colloidal stability and may support controlled selenium release, thereby improving bioavailability at the infection site. The pronounced activity against Candida albicans with a 34 mm inhibition zone may reflect enhanced fungal internalization, whereas the resistance observed in Pseudomonas aeruginosa is likely associated with efficient efflux pumps and ROS-scavenging systems.

### 3.5. Cytotoxic Activity of HA and Se/HA NPs

#### 3.5.1. Concentration-Dependent Cytotoxicity of HA and Se/HA NPs Against MG-63 Osteosarcoma Cells

**Figure 6:**
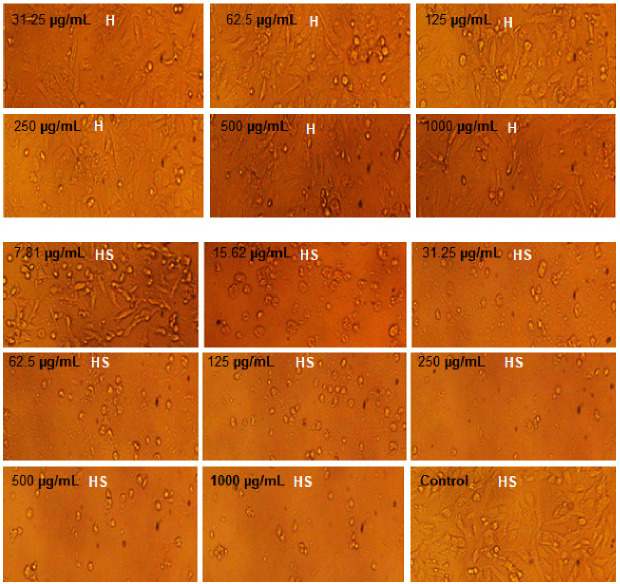
Morphological analysis of MG-63 osteosarcoma cells after 48 h of treatment with different concentrations of hyaluronic acid (HA; left panels) and selenium–hyaluronic acid nanoparticles (Se/HA NPs; right panels). Untreated control cells are shown in the top-left panel, 0 µg/mL, for both series. HA alone, shown in the left panels, caused no clear morphological changes even at high concentrations up to 1000 µg/mL, whereas Se/HA NPs, shown in the right panels, induced concentration-dependent cell shrinkage, rounding, and detachment starting from 7.81 µg/mL. Scale bar = 100 µm.

The cytotoxic effects of HA and Se/HA NPs against MG-63 osteosarcoma cells were evaluated across a broad concentration range of 0–1000 µg/mL (Table 2 and Figure 6). Both treatments showed no cytotoxicity at baseline concentration (0 µg/mL), confirming normal cell viability under untreated conditions. However, a clear difference was observed between HA and Se/HA NPs as the concentration increased. HA alone showed minimal cytotoxicity at low concentrations, with no detectable effect up to 15.62 µg/mL and only slight cytotoxicity between 31.25 and 125 µg/mL, where cell death remained ≤1.51%. At higher concentrations, HA induced only moderate cytotoxicity, reaching 12.87% at 250 µg/mL and increasing to 47.83% and 55.30% at 500 and 1000 µg/mL, respectively. These findings suggest that HA is largely biocompatible and exerts cytotoxic effects only at relatively high concentrations. In contrast, Se/HA NPs displayed a strong and concentration-dependent cytotoxic effect even at low concentrations. Cytotoxicity reached 46.61% at 7.81 µg/mL and increased sharply to 91.94% at 15.62 µg/mL. From 31.25 µg/mL onward, Se/HA NPs produced very high cytotoxicity levels of approximately 96–97%, indicating near-complete inhibition of MG-63 cell viability across this concentration range. This difference was further confirmed by the IC_50_ values, where HA showed a high IC_50_ of 746.37 µg/mL, reflecting low cytotoxicity, whereas Se/HA NPs exhibited a markedly lower IC_50_ of 8.36 µg/mL, confirming their potent anticancer activity. The high IC_50_ value of HA, 746.37 µg/mL, is consistent with the known biocompatibility of hyaluronic acid, which exhibits minimal cytotoxicity even at elevated concentrations, as reported in previous studies [38,39]. In contrast, the low IC_50_ value of Se/HA NPs, 8.36 µg/mL, reflects the potent anticancer activity conferred by selenium incorporation. Overall, these results highlight the novelty of Se/HA NPs as a highly active nanoconjugate capable of producing strong anticancer effects at low concentrations while HA alone remains comparatively biocompatible.

#### 3.5.2. Morphological Evidence of Se/HA-Induced Cytotoxic Damage

Microscopic examination further supported the cytotoxicity results by revealing clear treatment-dependent morphological changes in MG-63 cells (Figure 6). Cells in the untreated control group displayed typical healthy morphology, characterized by a well-spread, elongated appearance and firm adherence to the culture surface, indicating normal viability and growth. In the HA-treated groups, most cells retained their normal morphology and attachment pattern across the tested concentrations of 31.25–1000 µg/mL, with only slight reductions in cell density at higher concentrations. Minor morphological alterations, including limited shrinkage and reduced confluency, were observed at the highest HA concentrations; however, the majority of cells remained structurally intact. These observations are consistent with the low cytotoxicity percentages recorded for HA (Table 2). In contrast, cells exposed to Se/HA NPs showed pronounced concentration-dependent morphological damage. At low concentrations of 7.81–15.62 µg/mL, cells began to lose their characteristic spindle-like morphology, became rounder, and showed weaker attachment. As the concentration increased from 31.25 to 125 µg/mL, a marked reduction in cell number, increased cellular shrinkage, and progressive detachment from the surface were observed. At higher concentrations of 250–1000 µg/mL, severe cytopathic changes became evident, including extensive cell rounding, membrane disruption, accumulation of cellular debris, and near-complete loss of viable cells. These morphological findings confirm that Se/HA NPs not only reduce MG-63 cell viability quantitatively but also induce visible structural deterioration, supporting their novelty as a bioactive selenium-HA nanoplatform with strong tumor cell-damaging capacity.

#### 3.5.3. HA-Mediated Functionalization as a Driver of Enhanced Anticancer Activity

The superior anticancer activity of Se/HA NPs may be attributed to the combined contribution of selenium bioactivity and HA-mediated stabilization and cellular interaction. Previous work by Ren et al. [15] demonstrated that hyaluronic acid-modified selenium nanoparticles showed greater anticancer efficacy than selenium alone, with tumor inhibition of approximately 47–49%, enhanced immune response, and reduced oxidative stress. This synergistic interaction may result from the ability of HA to regulate selenium transport and release, thereby improving therapeutic efficiency while reducing potential toxicity [19]. HA also plays an important role as a targeting and stabilizing agent because of its ability to interact with cell-surface receptors, particularly CD44, which are frequently overexpressed in several tumor cells. Through receptor-mediated uptake, HA-functionalized nanoparticles can accumulate more selectively in tumor cells, thereby enhancing the therapeutic action of selenium. In agreement with this concept, Vafadar et al. [20] reported that incorporating HA with copper and selenium improved the anticancer activity of nanocomposites by reducing crystallite size, increasing particle stability, and enhancing biological activity. Their HA-coated selenium-copper nanocomposite showed stronger anticancer effects against HepG2 liver cancer cells than conventional Se NPs, inducing cell death even at low doses [20]. Functionalized selenium-based systems have also been shown to inhibit cancer cell proliferation, migration, and invasion while promoting apoptosis, with improved efficacy compared with free drugs or non-targeted formulations. In this context, the present study extends previous findings by demonstrating that ascorbic acid-assisted Se/HA NPs exert potent cytotoxic activity against MG-63 osteosarcoma cells, emphasizing the novelty of combining green synthesis, HA surface functionalization, and selenium bioactivity to generate promising nanoconjugate for anticancer applications.

### 3.6. Se/HA Nanoparticles Promote Accelerated Wound Closure and Cell Migration

**Figure 7:**
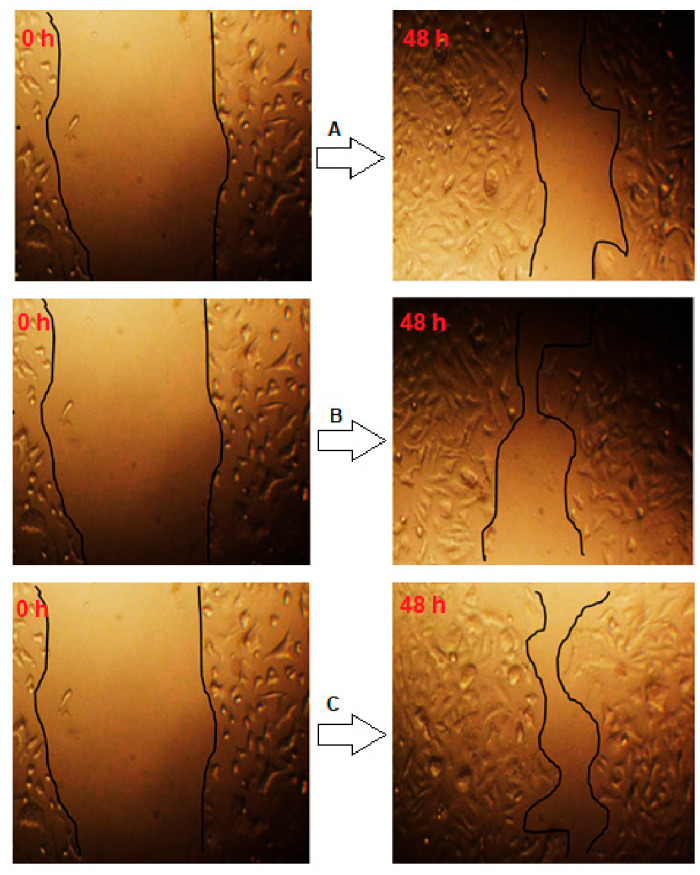
Scratch wound-healing assay of HFB4 cells: (**A**) untreated control cells, (**B**) cells treated with hyaluronic acid (HA), and (**C**) cells treated with selenium-hyaluronic acid nanoparticles (Se/HA NPs).

The wound-healing activity of HA and Se/HA NPs was evaluated using the scratch assay, and the results clearly demonstrated treatment-dependent enhancement of wound closure (Figure 7 and Table 3). At 0 h, all groups showed the same initial wound width (1022.43 µm) and wound area (1,099,771.72 µm^2^), confirming a uniform baseline and ensuring the validity of subsequent comparisons. After 48 h, distinct differences in wound closure became evident among the groups. In the untreated control group, the wound width decreased to 517.71 µm and the wound area to 556,922.7 µm^2^, corresponding to a wound closure percentage of 49.36% and an area reduction of 542,849.1 µm^2^. In the HA-treated group, wound repair was improved relative to the control, with the wound width decreasing further to 439.36 µm and the wound area to 472,703.3 µm^2^. This resulted in a wound closure percentage of 57.02% and a greater area reduction of 627,068.4 µm^2^, supporting the known role of HA in promoting cell migration, hydration, and tissue repair [38]. The most pronounced wound-healing effect was observed in the Se/HA NP-treated group. In this group, the wound width was markedly reduced to 271.87 µm and the wound area to 292,432.4 µm^2^, yielding the highest wound closure percentage of 73.41% and the largest area reduction of 807,339.3 µm^2^. In addition, the higher RM value of 15.64 µm/h further confirmed enhanced cell migration in response to Se/HA treatment (Table 3). These quantitative results were in agreement with the visual observations in Figure 7, which showed more extensive gap closure in the Se/HA-treated group than in the HA-treated and untreated groups. These findings are consistent with the report of Nejati et al. [39], who found that SeNPs doped with hyaluronic acid accelerated wound healing by combining the pro-migratory and tissue-supportive properties of HA with the antioxidant and antimicrobial effects of selenium nanoparticles.

The enhanced wound-healing efficacy of Se/HA NPs, reflected by 73.41% wound closure at 48 h, was observed at a concentration of 31.25 µg/mL. This concentration was selected based on preliminary cytotoxicity assays showing no adverse effects on HFB4 cell viability at this concentration. The mechanism underlying this effect is multifactorial. First, HA itself promotes cell migration by interacting with CD44 receptors on fibroblast membranes, activating downstream signaling pathways such as Rho GTPases and focal adhesion kinase (FAK), which regulate cytoskeletal reorganization and cell motility [38]. Second, selenium nanoparticles may contribute by reducing oxidative stress at the wound site through antioxidant enzyme-mimetic activity, such as glutathione peroxidase-like activity, thereby protecting migrating fibroblasts from oxidative damage [39]. Third, the anti-inflammatory activity of Se/HA NPs, with an IC_50_ of 5.37 µg/mL, may reduce local inflammation and create a more favorable microenvironment for tissue repair.

Comparisons with previous studies further support our findings. Nejati et al. [39] reported that Se NPs incorporated into HA-methacrylate/gelatin methacrylate hydrogels achieved approximately 65–70% wound closure in vitro after 48 h, which is comparable to our results. Hakki et al. [38] demonstrated that HA alone enhanced cell migration in cementoblasts, but the addition of selenium accelerated closure rates by an additional 15–20%. Our Se/HA NPs achieved 73.41% closure, exceeding the 57.02% closure observed with HA alone and the 49.36% closure observed in untreated controls, confirming the synergistic effect of Se and HA. Notably, Li et al. [36] recently reported a carboxymethyl chitosan/oxidized hyaluronic acid hydrogel containing Se NPs that achieved 96% closure in diabetic wound models after 14 days, suggesting that our Se/HA NPs could be further optimized for chronic wound applications. Collectively, the present data demonstrate that Se/HA NPs provide a stronger wound-healing response than HA alone, highlighting the novelty of this multifunctional nanoconjugate as a promising bioactive system for enhancing fibroblast migration and tissue repair.

### 3.7. Selenium Functionalization Enhances the Anti-Inflammatory Activity of HA

**Figure 8:**
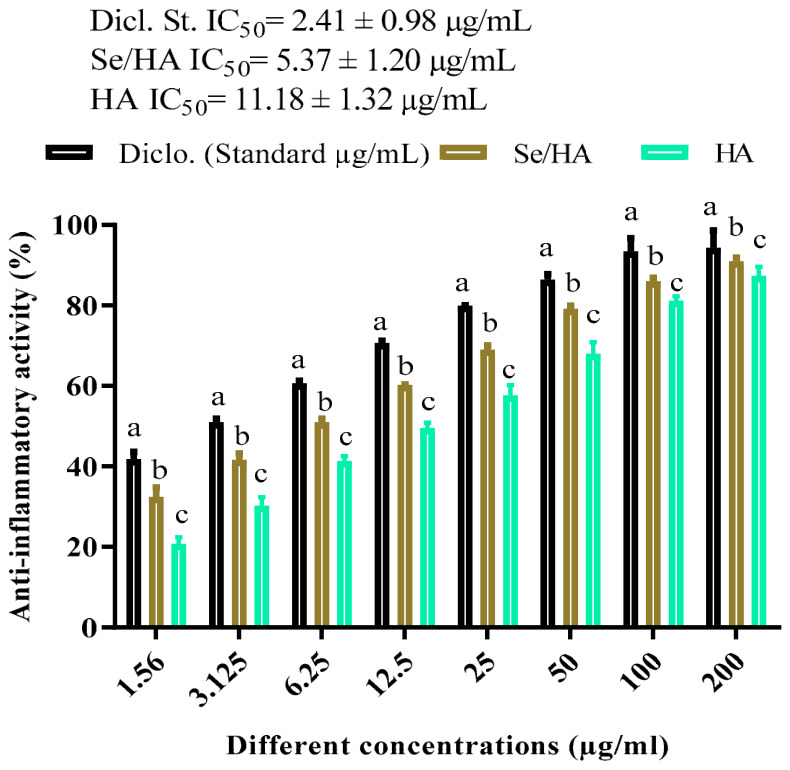
Anti-inflammatory activity, based on protein denaturation inhibition, of hyaluronic acid (HA) and selenium/HA nanoparticles (Se/HA NPs). Data are presented as mean ± SD. Different letters above the columns indicate statistically significant differences at *p* < 0.05.

The anti-inflammatory activity of HA and Se/HA NPs was evaluated in comparison with the standard drug diclofenac sodium using the protein denaturation inhibition assay (Figure 8). At lower concentrations of 1.56–6.25 µg/mL, diclofenac sodium showed the highest inhibitory activity, followed by Se/HA NPs, whereas HA exhibited the lowest effect. This pattern indicates that selenium incorporation markedly enhanced the anti-inflammatory performance of HA. A similar trend was observed at intermediate concentrations of 12.5–50 µg/mL, where Se/HA NPs consistently demonstrated significantly higher inhibition of protein denaturation than HA alone, confirming their improved anti-inflammatory potential. At higher concentrations of 100–200 µg/mL, both HA and Se/HA NPs approached strong inhibition levels; however, Se/HA NPs remained superior to HA. Although the difference between HA and Se/HA became statistically insignificant at 200 µg/mL, both treatments showed slightly lower activity than diclofenac sodium. The IC_50_ values further supported these findings, with diclofenac sodium showing the lowest IC_50_ value of 2.41 µg/mL, followed by Se/HA NPs at 5.37 µg/mL and HA at 11.18 µg/mL. These results confirm that Se/HA NPs possess substantially stronger anti-inflammatory efficiency than HA alone. The enhanced activity of Se/HA NPs may be attributed to the combined antioxidant properties of selenium and the biological functionality of HA, which together may reduce reactive oxygen species production and suppress inflammatory mediators, including pro-inflammatory cytokines [40].

The anti-inflammatory activity of HA alone, with an IC_50_ of 11.18 µg/mL, is consistent with the known properties of high-molecular-weight HA (HMW-HA, >1 MDa), which has been reported to exhibit anti-inflammatory effects by inhibiting cytokine production and blocking CD44-mediated pro-inflammatory signaling [37]. In contrast, low-molecular-weight HA (LMW-HA, <500 kDa) is often associated with pro-inflammatory responses. The use of HMW-HA in this study explains the observed anti-inflammatory activity of pure HA and supports its selection as a biocompatible, anti-inflammatory coating for Se NPs.

Overall, these findings demonstrate that selenium functionalization significantly improves the anti-inflammatory potential of HA, producing an activity profile that approaches that of diclofenac sodium and highlighting the novelty of Se/HA NPs as a multifunctional nanoconjugate with promising anti-inflammatory and tissue-protective properties.

### 3.8. Study Limitations

This study has several limitations that should be acknowledged. First, biological evaluations were primarily performed using in vitro models, which cannot fully reproduce the complexity of in vivo physiological conditions, including immune responses, tissue distribution, metabolism, and systemic clearance. Therefore, the promising antimicrobial, anticancer, anti-inflammatory, and wound-healing activities observed in this study should be further validated using appropriate in vivo models. Second, the long-term toxicity, pharmacokinetic behavior, biodistribution, and possible tissue accumulation of Se/HA nanoparticles were not investigated. These assessments are essential to determine their safety profile and translational suitability. Third, although the present findings demonstrate enhanced biological performance after HA-mediated selenium nano-conjugation, the precise molecular mechanisms responsible for these effects remain to be fully clarified. Fourth, this study did not include bare selenium nanoparticles, without HA coating, as a control for the anticancer assays. Therefore, while Se/HA NPs show potent cytotoxicity, with an IC_50_ of 8.36 µg/mL, we cannot definitively attribute the enhanced effect to HA modification rather than to the intrinsic cytotoxicity of selenium itself. It is possible that bare Se NPs may exhibit similar or even lower IC_50_ values. A direct comparison with empty Se NPs synthesized under identical conditions is necessary to quantify the contribution of HA conjugation. Further studies are required to examine cellular uptake pathways, receptor-mediated interactions, oxidative stress modulation, apoptosis-related signaling, inflammatory cytokine regulation, and nanoparticle–cell interactions. Additionally, this study did not include “empty” selenium nanoparticles, without HA coating, as a control. Such a control would help distinguish the contribution of HA from that of selenium itself to the observed biological activities. Future studies should synthesize bare Se NPs under identical conditions to enable direct comparison and confirm the synergistic role of HA. Furthermore, although the 30-day stability assessment demonstrates good short-term colloidal stability, longer-term stability beyond 30 days was not evaluated. Future studies should investigate stability over 6–12 months under various storage conditions, including lyophilized versus liquid formulations, different temperatures, and buffered media, to support potential clinical translation. Furthermore, the economic feasibility and scalability of the proposed Se/HA NPs should be considered. Although selenium is relatively abundant as a trace element, high-purity sodium selenite (Na_2_SeO_3_) and hyaluronic acid can be costly, particularly for large-scale production. The green synthesis method described herein uses ascorbic acid, vitamin C, as a reducing agent, which is inexpensive and widely available. However, the overall cost may still be higher than that of conventional antibiotics or antiseptics. Future studies should explore alternative selenium sources, such as selenium-rich plant extracts or industrial byproducts, to reduce costs while maintaining bioactivity. Additionally, the availability of pharmaceutical-grade HA may vary by region, potentially limiting clinical translation in resource-limited settings. Despite these challenges, the multifunctional nature of Se/HA NPs, combining anticancer, antimicrobial, anti-inflammatory, and wound-healing activities in a single formulation, may offset the higher upfront cost by replacing multiple therapeutic agents. Addressing these limitations in future work will strengthen the biological interpretation of Se/HA nanoparticles and support their development as a safe and effective multifunctional biomedical platform.

## 4. Conclusions

Se/HA nanoparticles were successfully prepared using an eco-friendly aqueous ascorbic acid-mediated synthesis approach. TEM analysis revealed well-dispersed, monodisperse spherical nanoparticles with particle sizes ranging from 29.72 to 80.38 nm, while XRD confirmed their crystalline nature through characteristic selenium diffraction peaks. The average crystallite size, calculated using the Scherrer equation, was 31.2 nm, further confirming the nanoscale structure of the synthesized Se/HA nanoparticles. Compared with unmodified HA, Se/HA nanoparticles demonstrated a clear enhancement in biological activity across all evaluated assays. Antimicrobial screening showed strong inhibition against *Enterococcus faecalis* (21 mm), *Staphylococcus aureus* (24 mm), *Escherichia coli* (25 mm), and *Klebsiella pneumoniae* (27 mm), together with marked antifungal activity against *Candida albicans* (34 mm), with an MIC value of 7.8 µg/mL. In the anticancer assay, Se/HA nanoparticles induced potent cytotoxicity against MG-63 osteosarcoma cells, as reflected by a low IC_50_ value of 8.36 µg/mL, whereas HA alone showed limited cytotoxicity with an IC_50_ value of 746.37 µg/mL. Moreover, Se/HA nanoparticles significantly enhanced wound closure, achieving 73.41% closure compared with 57.02% for HA, indicating improved cell migration and tissue-repair potential. The anti-inflammatory assay also confirmed the superior performance of Se/HA nanoparticles, with an IC_50_ value of 5.37 µg/mL, approaching the activity of diclofenac sodium (2.41 µg/mL). Collectively, these findings demonstrate that HA-mediated selenium nano-conjugation markedly improves antimicrobial, antifungal, anticancer, anti-inflammatory, and wound-healing activities. The novelty of this study lies in the development of a green, HA-stabilized selenium nanoplatform with integrated multifunctional bioactivity, supporting its potential as a promising candidate for biomedical applications, particularly in infection control, cancer therapy, inflammation modulation, and tissue repair. These findings introduce Se/HA NPs as a green multifunctional nanoplatform with promising anticancer, antimicrobial, anti-inflammatory, and wound-healing potential.

## Figures and Tables

**Table 1:** Antimicrobial activity of hyaluronic acid (HA) and selenium-hyaluronic acid nanoparticles (Se/HA NPs) against selected microorganisms, expressed as inhibition zone diameter, minimum inhibitory concentration, minimum bactericidal concentration, and minimum fungicidal concentration. Values are presented as mean ± standard deviation. Different superscript letters within the same row indicate statistically significant differences (*p* ≤ 0.05).

Examined Microorganisms	Inhibition Zones (mm)			MIC		MBC	
	HA	Se-HA	Standard	HA	Se/HA	HA	Se/HA
*E. faecalis*	14 ± 0.4 ^c^	21 ± 1.0 ^a^	18 ± 0.5 ^b^	62.5	15.62	500	31.25
*S. aureus*	21 ± 0.8 ^b^	24 ± 0.9 ^a^	23 ± 0.3 ^a^	31.25	15.62	125	31.25
*E. coli*	22 ± 0.2 ^b^	25 ± 0.5 ^a^	25 ± 0.5 ^a^	31.25	15.62	125	31.25
*K. pneumoniae*	23 ± 0.9 ^b^	27 ± 0.2 ^a^	24 ± 0.2 ^b^	15.62	15.62	125	31.25
*P. aeruginosa*	NA	15 ± 0.8 ^b^	18 ± 0.7 ^a^	----	250	----	1000
*C. albicans*	23 ± 0.1 ^b^	34 ± 0.8 ^a^	22 ± 0.4 ^b^	15.62	7.8	125	15.62

**Table 2:** Cytotoxicity percentage of hyaluronic acid (HA) and selenium-hyaluronic acid nanoparticles (Se/HA NPs) against MG-63 osteosarcoma cells. Different superscript letters within the same row indicate statistically significant differences (*p* ≤ 0.05).

Concentration (µg/mL)	Cytotoxicity (%)	
	HA	Se/HA
0	0.0	0.0
7.81	0.0 ^b^	46.61 ± 0.02 ^a^
15.62	0.0 ^b^	91.94 ± 0.25 ^a^
31.25	0.41 ± 0.03 ^b^	96.23 ± 0.07 ^a^
62.5	0.70 ± 0.16 ^b^	96.70 ± 1.32 ^a^
125	1.51 ± 0.15 ^b^	96.64 ± 0.52 ^a^
250	12.87 ± 1.06 ^b^	96.87 ± 1.65 ^a^
500	47.83 ± 1.36 ^b^	96.81 ± 1.87 ^a^
1000	55.30 ± 1.25 ^b^	96.812 ± 1.32 ^a^
IC_50_ (µg/mL)	746.37 ± 2.79 ^a^	8.36 ± 0.39 ^b^

**Table 3:** Wound-healing activity of hyaluronic acid (HA) and selenium-hyaluronic acid nanoparticles (Se/HA NPs), expressed as wound width, wound area, wound closure percentage, area reduction, and rate of migration.

Treatment	At 0 h		At 48 h		RM um	Wound Closure % um^2^	Area Difference
	Mean Width	Mean Area	Mean Width	Mean Area			
Control	1022.43	1,099,771.72	517.71	556,922.7	10.51	49.36	542,849.1
HA	1022.43	1,099,771.72	439.36	472,703.3	12.15	57.02	627,068.4
Se/HA NPs	1022.43	1,099,771.72	271.87	292,432.4	15.64	73.41	807,339.3

## Data Availability

The original contributions presented in this study are included in the article/Supplementary Material. Further inquiries can be directed to the corresponding author.

## Supplementary Materials

The following supporting information can be downloaded at: https://www.mdpi.com/article/10.3390/polym18111376/s1, Table S1: Time-dependent colloidal stability parameters of Se/HA NPs stored at 4 °C for 30 days.

## References

[1] Malik S., Muhammad K., Waheed Y. (2023). Emerging Applications of Nanotechnology in Healthcare and Medicine. Molecules.

[2] Kudarha R., Colaco V., Gupta A., Kulkarni S., Soman S., Kulkarni J., Rana K., Navti P., Tiwari R., Osmani R. (2024). Recent advancements in selenium nanoconstructs as a potential carrier in cancer therapy. Nano-Struct. Nano-Objects.

[3] Burlec A.F., Corciova A., Boev M., Batir-Marin D., Mircea C., Cioanca O., Danila G., Danila M., Bucur A.F., Hancianu M. (2023). Current Overview of Metal Nanoparticles’ Synthesis, Characterization, and Biomedical Applications, with a Focus on Silver and Gold Nanoparticles. Pharmaceuticals.

[4] Yahya R., Al-Rajhi A.M.H., Alzaid S.Z., Al Abboud M.A., Almuhayawi M.S., Al Jaouni S.K., Selim S., Ismail K.S., Abdelghany T.M. (2022). Molecular Docking and Efficacy of Aloe vera Gel Based on Chitosan Nanoparticles against Helicobacter pylori and Its Antioxidant and Anti-Inflammatory Activities. Polymers.

[5] Abdelghany T.M., Al-Rajhi A.M.H., Almuhayawi M.S., Abada E., Al Abboud M.A., Moawad H., Yahya R., Selim S. (2023). Green fabrication of nanocomposite doped with selenium nanoparticle–based starch and glycogen with its therapeutic activity: Antimicrobial, antioxidant, and anti-inflammatory in vitro. Biomass Convers. Biorefinery.

[6] Alghonaim M.I., Alsalamah S.A., Mohammad A.M., Abdelghany T.M. (2025). Green synthesis of bimetallic Se@TiO2NPs and their formulation into biopolymers and their utilization as antimicrobial, anti-diabetic, antioxidant, and healing agent in vitro. Biomass Convers. Biorefinery.

[7] Al-Rajhi A.M.H., Alsalamah S.A., Alruhaili M.H., Gattan H.S., Alharbi M.T., Kafy S.M., Selim S., Qanash H., Bazaid A.S., Abdelghany T.M. (2025). Innovative vaginal wash formulation with Chitosan nanoparticles targets microbial pathogens, ovarian cancer and inflammation. Sci. Rep..

[8] Karthik K.K., Cheriyan B.V., Rajeshkumar S., Gopalakrishnan M. (2024). A review on selenium nanoparticles and their biomedical applications. Biomed. Technol..

[9] Amin M.A., Algamdi N.A., Waznah M.S., Bukhari D.A., Alsharif S.M., Alkhayri F., Abdel-Nasser M., Fouda A. (2024). An Insight into Antimicrobial, Antioxidant, Anticancer, and Antidiabetic Activities of Trimetallic Se/ZnO/CuO Nanoalloys Fabricated by Aqueous Extract of Nitraria retusa. J. Clust. Sci..

[10] Soliman M.K.Y., Amin M.A.-A., Nowwar A.I., Hendy M.H., Salem S.S. (2024). Green synthesis of selenium nanoparticles from Cassia javanica flowers extract and their medical and agricultural applications. Sci. Rep..

[11] Chauhan N.P.S., Derakhshani A., Jain K., Chittora A., Soni D., Malik A., Marwal A., Jadoun S. (2026). Recent advances in the green synthesis of metal and metal oxide nanoparticles from plant extracts for biomedical and water remediation applications. Next Nanotechnol..

[12] Shahabadi N., Zendehcheshm S., Khademi F. (2021). Selenium nanoparticles: Synthesis, in-vitro cytotoxicity, antioxidant activity and interaction studies with ct-DNA and HSA, HHb and Cyt c serum proteins. Biotechnol. Rep..

[13] El-Saadony M.T., Saad A.M., Alkafaas S.S., Dladla M., Ghosh S., Mohammed D.M., Soliman T.N., Elkelish A., Khalil F.M.A., Fahmy M.A. (2026). Selenium nanoparticles: Eco-friendly synthesis, biological activities and biomedical applications—A comprehensive review. Mater. Today Bio.

[14] Geng L., Li L., Sun X., Cheng S., He J. (2025). Recent Advances Towards Selenium Nanoparticles: Synthetic Methods, Functional Mechanisms, and Biological Applications. Foods.

[15] Ren Y., Zhao T., Mao G., Zhang M., Li F., Zou Y., Yang L., Wu X. (2013). Antitumor activity of hyaluronic acid–selenium nanoparticles in Heps tumor mice models. Int. J. Biol. Macromol..

[16] Xia Y., Guo M., Xu T., Li Y., Wang C., Lin Z., Zhao M., Zhu B. (2018). siRNA-loaded selenium nanoparticle modified with hyaluronic acid for enhanced hepatocellular carcinoma therapy. Int. J. Nanomed..

[17] Iaconisi G.N., Lunetti P., Gallo N., Cappello A.R., Fiermonte G., Dolce V., Capobianco L. (2023). Hyaluronic Acid: A Powerful Biomolecule with Wide-Ranging Applications-A Comprehensive Review. Int. J. Mol. Sci..

[18] Misra S., Hascall V.C., Markwald R.R., Ghatak S. (2015). Interactions between Hyaluronan and Its Receptors (CD44, RHAMM) Regulate the Activities of Inflammation and Cancer. Front. Immunol..

[19] Luo W., Li Y., Zhao J., Niu R., Xiang C., Zhang M., Xiao C., Liu W., Gu R. (2024). CD44-targeting hyaluronic acid-selenium nanoparticles boost functional recovery following spinal cord injury. J. Nanobiotechnology.

[20] Vafadar M.S., Ghabool Y., Ahmadi K., Sareban N., Reghbati M., Behmadi H., Zare-Zardini H., Nematollahi A., Behboodian B., Es-haghi A. (2025). Selenium-copper hyaluronic acid nanoparticles: A novel green synthesis for improved anticancer activity against HepG2 liver cancer cells. Inorg. Chem. Commun..

[21] Selim S., Saddiq A.A., Ashy R.A., Baghdadi A.M., Alzahrani A.J., Mostafa E.M., Al Jaouni S.K., Elamir M.Y.M., Amin M.A., Salah A.M. (2025). Bimetallic selenium/zinc oxide nanoparticles: Biological activity and plant biostimulant properties. AMB Express.

[22] Abdelghany T.M., Yahya R., Bakri M.M., Ganash M., Amin B.H., Qanash H. (2021). Effect of Thevetia peruviana seeds extract for microbial pathogens and cancer control. Int. J. Pharmacol..

[23] Zimmer B.L., Carpenter D.E., Esparza G., Alby K., Bhatnagar A., Ferrell A.L., Flemming L., Huband M.D., Jiménez-Pearson A., Kircher S.M. (2024). CLSI M07, Methods for Dilution Antimicrobial Susceptibility Tests for Bacteria That Grow Aerobically. https://cdn.bfldr.com/YLD4EVFU/at/ff6qbv6bxmgzmg9vwb9fgkc/m07ed12e_sample.pdf.

[24] (2019). Susceptibility Testing of Infectious Agents and Evaluation of Performance of Antimicrobial Susceptibility Test Devices, Part 1: Broth Micro-Dilution Reference Method for Testing the In Vitro Activity of Antimicrobial Agents Against Rapidly Growing Aerobic Bacteria Involved in Infectious Diseases, 2nd Edition.

[25] Almehayawi M.S., Almuhayawi M.S., El-Fadl S.R.A., Nagshabandi M.K., Tarabulsi M.K., Selim S., Alruwaili Y.S., Mostafa E.M., Al Jaouni S.K., Abdelghany T.M. (2024). Evaluating the anti-yeast, anti-diabetic, wound healing activities of Moringa oleifera extracted at different conditions of pressure via supercritical fluid extraction. BioResources.

[26] Alsalamah S.A., Alghonaim M.I., Jusstaniah M., Abdelghany T.M. (2023). Anti-Yeasts, Antioxidant and Healing Properties of Henna Pre-Treated by Moist Heat and Molecular Docking of Its Major Constituents, Chlorogenic and Ellagic Acids, with Candida albicans and Geotrichum candidum Proteins. Life.

[27] Alawlaqi M.M., Al-Rajhi A.M.H., Abdelghany T.M., Ganash M., Moawad H. (2023). Evaluation of Biomedical Applications for Linseed Extract: Antimicrobial, Antioxidant, Anti-Diabetic, and Anti-Inflammatory Activities In Vitro. J. Funct. Biomater..

[28] Zhang Y., Wang J., Zhang L. (2010). Creation of highly stable selenium nanoparticles capped with hyperbranched polysaccharide in water. Langmuir.

[29] Zhang X., Yan H., Ma L., Zhang H., Ren D.-F. (2020). Preparation and characterization of selenium nanoparticles decorated by Spirulina platensis polysaccharide. J. Food Biochem..

[30] Li J., Zhou D., Liu Y., Chen Y., Chen J., Yang Y., Gao Y., Qiu J. (2023). Engineering CsPbX3 (X = Cl, Br, I) Quantum Dot-Embedded Borosilicate Glass through Self-Crystallization Facilitated by NaF as a Phosphor for Full-Color Illumination and Laser-Driven Projection Displays. ACS Appl. Mater. Interfaces.

[31] Coelho D., Luiz G.M., Machado S.A.S. (2018). A photoelectrochemical methodology to obtain nanorods of crystalline hexagonal trigonal selenium. J. Electroanal. Chem..

[32] El-Sayed M.H., Shubaily H.M., Abdelglil M.I., Alenazi N., Salama S.A., Abdel-Khalek E.K., Sharaf M.H., Amin M.A.-A. (2025). Green Synthesis of CuO and Se Nanoparticles and CuO/Se Agglomerates of NPs by Anabasis setifera Biomass Extract: Antimicrobial, Antioxidant, Antibiofilm, and Anticancer Activities. BioResources.

[33] Pan N.C., Pereira H.C.B., da Silva M.L.C., Vasconcelos A.F.D., Celligoi M. (2017). Improvement Production of Hyaluronic Acid by Streptococcus zooepidemicus in Sugarcane Molasses. Appl. Biochem. Biotechnol..

[34] Mohammed A.A., Niamah A.K. (2022). Identification and antioxidant activity of hyaluronic acid extracted from local isolates of Streptococcus thermophilus. Mater. Today Proc..

[35] Zhang C., Song J., Shen X., Li Q., Su F., Li S. (2023). Fluorescent nanoprobe prepared from hyaluronic acid modified iron selenide nanoparticles for real-time detection of hyaluronidase as tumor marker. J. Pharm. Biomed. Anal. Open.

[36] Li W.-X., Li S., Cheng H., Chen Y., Hu H.-J., Tang S., Sun J., Liao X.-P., Jiang G.-B., Zhang H. (2026). A glucose-triggered self-reinforcing hydrogel based on carboxymethyl chitosan/oxidized hyaluronic acid/borax for delivering nano-selenium and dynamic diabetic wound management. Carbohydr. Polym..

[37] Altman R., Bedi A., Manjoo A., Niazi F., Shaw P., Mease P. (2019). Anti-Inflammatory Effects of Intra-Articular Hyaluronic Acid: A Systematic Review. Cartilage.

[38] Hakki S.S., Bozkurt S.B., Sculean A., Božić D. (2024). Hyaluronic acid enhances cell migration, viability, and mineralized tissue-specific genes in cementoblasts. J. Periodontal Res..

[39] Nejati O., Tışlı B., Yaşayan G., Zaman B.T., Torkay G., Dönmez M., Kayın İ., Bakırdere S., Bal-Öztürk A. (2024). Microwave-assisted hydrothermal green synthesis of selenium nanoparticles incorporated with hyaluronic acid methacrylate/gelatin methacrylate hydrogels for wound healing applications. Polym. Eng. Sci..

[40] Zhuo M., Cheng N., Du J., Wang T., Sun C., Lu S., Wang J., Ding D. (2025). Selenium-functionalized hyaluronic acid dissolvable microneedle patch synergistic herbal shikonin-loaded tetrahedral framework nucleic acids for psoriasis therapy. Mater. Today Bio.

