# Supplementary material for: Eco-Friendly Selenium-Hyaluronic Acid Nanoconjugates with Potent Anticancer, Antimicrobial, Anti-Inflammatory and Wound-Healing Activities
Source: Polymers (Basel). 2026 Jun 1;18(11):1376. doi: 10.3390/polym18111376 (PMC13258865; doi:10.3390/polym18111376)
Supplement: Supplementary file 1 [file polymers-18-01376-s001.zip › polymers-4332514-supplementary.pdf]

Article

Eco-Friendly Selenium-Hyaluronic Acid Nanoconjugates with Potent Anticancer, Antimicrobial, Anti-inflammatory and Wound-Healing Activities

Husam Qanash [1,2,*], Bandar Alharbi [1,2], Abdulrahman S. Bazaid [1,2], Ghaida Alsaif [1,2], Talal Alharazi [1,2] and Naif K. Binsaleh [1,2]

[1] Department of Medical Laboratory Science, College of Applied Medical Sciences, University of Ha'il, Hail 55476, Saudi Arabia
[2] Medical and Diagnostic Research Center, University of Ha'il, Hail 55473, Saudi Arabia
* Correspondence: h.qanash@uoh.edu.sa (H.Q.)

Supplementary Table 1: Time-dependent colloidal stability parameters of Se/HA NPs stored at 4 °C for 30 days.

Time (days)	Diameter (nm)	PDI	Zeta Potential (mV)
**0**	68.4 ± 4.2 [a]	0.21 ± 0.03 [a]	−32.7 ± 2.8 [a]
**7**	69.1 ± 5.0 [a]	0.22 ± 0.04 [a]	−31.9 ± 3.1 [a]
**14**	72.3 ± 5.8 [a,b]	0.24 ± 0.05 [a,b]	−30.5 ± 3.4 [a,b]
**21**	78.6 ± 6.5 [b]	0.28 ± 0.06 [b]	−28.1 ± 3.9 [b]
**30**	89.2 ± 8.1 [c]	0.34 ± 0.07 [c]	−25.4 ± 4.2 [b,c]

Values are mean ± SD ($n = 3$). Different superscript letters indicate significant differences compared to day 0 ($p \leq 0.05$).
